# A robust deep learning approach for segmenting cortical and trabecular bone from 3D high resolution µCT scans of mouse bone

**DOI:** 10.1038/s41598-025-92954-1

**Published:** 2025-03-13

**Authors:** Amine Lagzouli, Peter Pivonka, David M. L. Cooper, Vittorio Sansalone, Alice Othmani

**Affiliations:** 1 School of Mechanical, Medical, and Process Engineering, Queensland University of Technology, Brisbane, Australia; 2 Univ Paris Est Créteil, Univ Gustave Eiffel, CNRS, UMR 8208, MSME, F-94010 Créteil, France; 3 Department of Anatomy, Physiology, and Pharmacology, University of Saskatchewan, Saskatoon, SK Canada; 4 LISSI, Université Paris-Est Creteil (UPEC), 94400 Vitry sur Seine, France

**Keywords:** 3D image segmentation, Deep learning, Microcomputed tomography µCT, High-resolution, Mouse tibia, Robust model, Computer science, Medical imaging, Biomedical engineering, Preclinical research

## Abstract

Recent advancements in deep learning have significantly enhanced the segmentation of high-resolution microcomputed tomography (µCT) bone scans. In this paper, we present the dual-branch attention-based hybrid network (DBAHNet), a deep learning architecture designed for automatically segmenting the cortical and trabecular compartments in 3D µCT scans of mouse tibiae. DBAHNet’s hierarchical structure combines transformers and convolutional neural networks to capture long-range dependencies and local features for improved contextual representation. We trained DBAHNet on a limited dataset of 3D µCT scans of mouse tibiae and evaluated its performance on a diverse dataset collected from seven different research studies. This evaluation covered variations in resolutions, ages, mouse strains, drug treatments, surgical procedures, and mechanical loading. DBAHNet demonstrated excellent performance, achieving high accuracy, particularly in challenging scenarios with significantly altered bone morphology. The model’s robustness and generalization capabilities were rigorously tested under diverse and unseen conditions, confirming its effectiveness in the automated segmentation of high-resolution µCT mouse tibia scans. Our findings highlight DBAHNet’s potential to provide reliable and accurate 3D µCT mouse tibia segmentation, thereby enhancing and accelerating preclinical bone studies in drug development. The model and code are available at https://github.com/bigfahma/DBAHNet.

## Introduction

Preclinical studies are essential for exploring the biological regulation of the musculoskeletal system. They are required in all drug development pipelines, where both small and large animal models are used to assess the efficacy and side effects of treatments on bone remodeling^1,2^. Bone remodeling is a dynamic, continuous process where old bone tissue is resorbed by osteoclasts and new bone tissue is formed by osteoblasts^3^. This cycle is influenced by mechanical loading, hormonal regulation, drug treatments, and diseases such as osteoporosis^4^. A precise understanding of how cortical and trabecular bones respond to these factors is crucial for biomechanical analysis, as it helps to better understand bone remodeling dynamics and aids in the development of new therapeutic drugs. Cortical bone provides structural support and strength, while trabecular bone, with its spongy structure, plays a key role in energy absorption and metabolic activities^5,6^. Therefore, segmenting these two regions separately is essential for accurately assessing the effects of various treatments and stimuli on the two different compartments.

Microcomputed tomography (µCT) is the gold standard for quantifying skeletal structure-function relationships, disease progression, and regeneration in preclinical models. With the use of µCT, major scientific advancements in osteoporosis, bone fracture healing, bone scaffold tissue engineering, and bone cancer metastasis have been made^7,8^. A major drawback of µCT is the acquisition of large volumes of data, which requires significant manual labor for processing and statistical analysis. Following data acquisition, the segmentation of bone structures is crucial for subsequent quantitative analysis. Manual segmentation is widely used but poses challenges due to its labor-intensive nature and potential for variability and bias among annotators^9^. This variability can result in inconsistencies across studies, impacting the generalization of findings^10^. The urgent requirement for automated segmentation processes is evident, aiming to increase both reproducibility and efficiency in bone analysis. Semiautomatic manual segmentation of large 3D µCT scans frequently uses interpolation algorithms^11^, segmenting sampled slices from the 3D scan and interpolating over the remaining slices. However, the effectiveness of this approach depends heavily on the sampling step size. A larger step size can lead to segmentation errors in interpolated slices, affecting the overall accuracy. It is essential to strike a balance, as decreasing the step size improves accuracy but incurs a higher time cost in the segmentation process.

Moreover, the literature reveals a notable gap concerning the development of a robust algorithm capable of accurately segmenting diverse types of bone scans. Conventional methods often struggle to generalize across various bone scans, underscoring the need for more adaptable and versatile segmentation approaches. While traditional automated segmentation algorithms utilizing classical morphological filtering and 3D image-processing techniques have proven effective^12,13^, their performance is often hindered by the morphological variations resulting from different experimental conditions. Despite their accuracy, segmentation techniques based on dual-thresholding of cortical and trabecular bone require calibration phantoms and precise voxel values for effective segmentation, leading to larger scan sizes. This increase necessitates significant computational resources for data storage and processing. Furthermore, these methods face difficulties in maintaining consistent volume segmentation quality in the presence of noise and varying recording conditions, affecting their ability to accurately segment bone structures and maintain connectivity. Segmenting cortical and trabecular bone is particularly challenging in the metaphysis region near the growth plate of long bones, where even experts find it difficult to manually delineate these structures owing to their complexity and intermingling. This complexity is exacerbated by various factors, such as drug treatments with anabolic or anticatabolic (or antiresorptive) effects, such as intermittent parathyroid hormone (PTH), risedronate, or mechanical loading (ML). These factors induce significant bone remodeling^14–16^ and significantly complicate the segmentation process.

Deep learning has revolutionized biomedical imaging segmentation, particularly in high-resolution µCT scans of bones^17–20^. Models based on deep learning, especially convolutional neural networks (CNNs), have demonstrated exceptional capability in automatically segmenting medical images with remarkable accuracy and speed. This success is attributed to the ability of CNNs to capture local features and dependencies between voxels, optimizing filters to detect and recognize relevant features effectively. These advancements in deep-learning-based image analysis techniques have the potential to automate µCT data processing, providing rapid research outcomes. Neeteson et al. developed and validated a fully automated segmentation algorithm for human high-resolution peripheral quantitative computed tomography (HR-pQCT) images of the distal radius and tibia, employing a U-Net-based architecture^21^. This method achieved high precision, even in images with significant cortical porosity. Similarly, Klein et al. introduced a dependable, fully automated method for bone segmentation in whole-body CT scans of patients with multiple myeloma, utilizing a U-Net-based framework^22^. Schoppe et al. presented a deep learning pipeline called AIMOS^23^, comprising a preprocessing module, a deep learning backbone, and a postprocessing module that automatically segments major organs in whole-body mouse scans^24^. Malimban et al. (2022) demonstrated that 3D models of nnU-Net^25^ achieve superior segmentation accuracy and are more robust to unseen data than 2D models^26^. They also compared the performance of nnU-Net with that of AIMOS on µCT images of the thorax in mouse µCT images. Integrating robust hybrid neural network architectures into the nnUNet framework has proven to work better for medical imaging, as seen with nnFormer^27^.

Since their popular launch in the natural language processing field, attention mechanisms and transformers have become prevalent in computer vision^28,29^. Self-attention has proven to be highly effective for developing neural network architectures for image processing. Oktay et al. introduced attention gates, which filter feature maps generated in the encoder and transmitted through skip connections to the decoder^30^. Transformers are increasingly popular in computer vision tasks because of their ability to capture long-range dependencies within a 3D scan. The Vision Transformer (ViT) was introduced in 2020^31^, marking the first fully transformer-based architecture to achieve state-of-the-art performance. To improve efficiency, Swin Transformers^32^ employ shifted windows for enhanced global attention by applying self-attention to nonoverlapping windows and enabling cross-window connections in subsequent layers. There is growing interest in hybrid architectures that combine transformers and CNNs, such as UNETR^33^ and SwinUNETR^34^. These hybrid architectures excel at capturing both the global and the local context within a 3D scan, providing a more comprehensive data representation.

Given the heterogeneity of bone imaging data sources and the variability introduced by different treatments, there is an urgent need to automate the segmentation process and ensure its generalization. Generalization is critical for applying models to unseen data and relies on strategies such as data augmentation to increase model robustness and adaptability^35^. Importantly, the ability to generalize is essential to ensuring that a model trained on limited data can accurately segment images under new biological conditions, scanned at different resolutions, and produced by different µCT devices. This capability ensures that the model maintains high accuracy and reliability across a wide array of preclinical scenarios, broadening its applicability in biomedical research, particularly in understanding bone adaptation processes and studying bone diseases, such as osteoporosis.

In this paper, we proposed a novel hybrid deep learning architecture, the Dual-Branch Attention-based Hybrid Network (DBAHNet)^36^, for high-resolution µCT mouse tibia image segmentation. DBAHNet yields excellent segmentation results on a diverse µCT mouse bone dataset, even when trained on a limited control set of high-resolution µCT (4.8–5 µm) mouse tibia images. Our objective was to develop a robust and generalizable model capable of accurately segmenting the cortical and trabecular compartments in high-resolution µCT images across various conditions. This model can be integrated into an automated pipeline for high-resolution µCT mouse bone assessment, automating the analysis in preclinical studies. This advancement is expected to increase our understanding of bone remodeling dynamics and the effects of drugs and mechanical loading on bones.

We collected a large dataset from seven different research studies^14–16,37–40^, encompassing various imaging resolutions (4.8–13.7 µm), mouse strains, and experimental conditions (drug treatments and mechanical loading). This highlighted the robustness and generalization capabilities of our deep learning architecture in handling unseen scenarios. Specifically, we trained our model on only 74 control mouse tibia µCT 3D scans and evaluated its performance across a large, diverse dataset. Our model achieved high accuracy in segmenting cortical and trabecular compartments and performed effectively in extreme cases where the bone shape deviated significantly from that of control bones, underscoring its ability to generalize from a limited dataset. Our findings suggest that DBAHNet can achieve robust and precise segmentation even in challenging and previously unseen scenarios. This capability is attributed to the neural network’s ability to effectively learn and differentiate the hidden features of the cortical and trabecular bone.

## Results

### Experimental design

The main training and experiments were conducted with 4 NVIDIA V100 32 GB GPUs. We employed the stochastic gradient descent (SGD) optimizer with a momentum of 0.99, a batch size of 4, and a cosine annealing learning rate scheduler starting at $1 \times 10^{-4}$. The 3D scans of the bone were each divided into 10 subsets along the z-axis (i.e., the long bone axis). This division is crucial because of the nature of the high-resolution data, as it reduces the size of the scans during data loading. The input scans were randomly cropped into subvolumes of size (320, 320, 32) and subjected to data augmentation and preprocessing. We evaluated the performance of our model via the Sørensen-Dice score coefficient (DSC) and the 95th percentile of the Hausdorff distance (HD95)^41^. The DSC measures the overlap between the predicted segmentation and the ground truth, which is calculated using Eq. (1).

1 $$\begin{aligned} \text {DSC} = \frac{2 \times |X \cap Y|}{|X| + |Y|} \end{aligned}$$

where $X$ is the set of predicted segmentation pixels and $Y$ is the set of ground truth segmentation pixels.

The Hausdorff distance (HD) measures the distance between two sets of points and is defined with Eq. (2).

2 $$\begin{aligned} d_H(X, Y) = \max \left\{ \sup _{x \in X} \inf _{y \in Y} d(x,y), \sup _{y \in Y} \inf _{x \in X} d(x,y) \right\} \end{aligned}$$

where $d(x,y)$ is the Euclidean distance between points $x$ and $y$. The 95th percentile of the Hausdorff distance (HD95) is used to mitigate the effect of outliers and is calculated as the 95th percentile of all the distances.

We used a combined Dice cross-entropy loss function for training, which combines the Dice loss and cross-entropy (CE) loss to optimize the model’s segmentation performance. The loss function is defined with Eq. (3).

3 $$\begin{aligned} \begin{aligned} \text {Dice Loss}&= 1 - \frac{2 \sum _{i=1}^{N} p_i g_i}{\sum _{i=1}^{N} p_i + \sum _{i=1}^{N} g_i} \\ \text {Cross-Entropy Loss}&= -\sum _{i=1}^{N} \left[ g_i \log (p_i) + (1 - g_i) \log (1 - p_i) \right] \\ \text {Loss}&= \alpha \times \text {Dice Loss} + \beta \times \text {Cross-Entropy Loss} \end{aligned} \end{aligned}$$

where $p_i$ is the predicted binary mask, $g_i$ is the ground truth binary mask, and $N$ is the total number of pixels. The weighting factors $\alpha$ and $\beta$ are both set to 0.5 to balance the contributions of the two loss components.

The numbers of attention heads used for the transformers at each hierarchical level were 6, 12, 24, and 48. The embedding dimension $C$ in the final model was set to $C = 96$. The main control dataset used for training and comparison with other state-of-the-art architectures was split into 70% for training, 10% for validation, and 20% for testing. We used the validation set to monitor the training and conduct the ablation study experiments, whereas the test set was kept isolated to evaluate the model’s performance against popular state-of-the-art architectures. For the ablation study and assessment of model complexity, we used the number of parameters (N Params) in millions and the giga floating point operations per second (GFLOPS).

### Performance comparison with other state-of-the-art models

The proposed DBAHNet model achieved state-of-the-art performance with an average DSC of 98.41%, a DSC of 99.13% for the cortical bone, and a DSC of 97.69% for the trabecular bone, prior to any post-processing. DBAHNet also demonstrated the best results in terms of boundary precision, with an average HD95 of 0.0095 mm, including 0.0080 mm for the cortical bone and 0.0110 mm for the trabecular bone.

As shown in Table 1, DBAHNet consistently outperformed other well-established architectures, including UNet, Attention UNet, UNETR, and SwinUNETR, across both the cortical and trabecular compartments. In addition to achieving the highest DSC scores, DBAHNet exhibited the lowest HD95 across all models. For instance, while SwinUNETR performed well with a DSC of 99.02% for the cortical compartment, its HD95 value of 0.0498 mm is notably higher than DBAHNet’s 0.0080 mm, highlighting DBAHNet’s superior segmentation accuracy and boundary precision.

**Table 1:** Performance comparison of the proposed method with state-of-the-art models on the control dataset^14–16^. The results include the average Dice Similarity Coefficient (DSC) and the average 95th percentile of Hausdorff Distance (HD95) for both cortical (C) and trabecular (T) compartments.

Models	Avg DSC	DSC C	DSC T	Avg HD95	HD95 C	HD95 T
UNet	0.9007	0.9049	0.8965	0.4129	0.4440	0.3818
Attention UNet	0.9507	0.9629	0.9384	0.1931	0.2013	0.1850
UNETR	0.9662	0.9832	0.9492	0.1131	0.1269	0.0992
SwinUNETR	0.9735	0.9902	0.9569	0.0498	0.0153	0.0843
DBAHNet	**0.9841**	**0.9913**	**0.9769**	**0.0095**	**0.0080**	**0.0110**

Best performances are in bold.

### Quantitative and qualitative segmentation results

We presented both quantitative and qualitative evaluations of the segmentation results obtained using DBAHNet in this section. The model was trained primarily on control datasets and tested on 3D high-resolution µCT scans of mouse tibiae under various medical treatments. We assessed performance separately for the cortical (C) and trabecular (T) compartments within the specified regions of interest.

#### Quantitative evaluation

We presented the segmentation results for our main datasets^14–16^ in Table 2. These results comprehensively demonstrated the model’s effectiveness in delineating bone structures under diverse and extreme drug treatments. The model exhibited robust performance under challenging conditions, including high doses of PTH and risedronate, as well as mechanical loading and their interactions, which significantly influenced bone remodelling and porosity.

**Table 2:** Quantitative evaluation of DBAHNet segmentation performance across various medical treatments for the main datasets. The results include the Dice similarity coefficient (DSC) and 95th percentile of the Hausdorff distance (HD95) for both the cortical (C) and the trabecular (T) compartments. $N_{\text {sub}}$ represents the total number of subsets, with each scan divided into 10 subsets along the z-axis.

Doses	Avg DSC	DSC C	DSC T	Avg HD95	HD95 C	HD95 T
Dataset 1^14^						
20 (µg/kg/day) ($N_{\text {sub}}$ = 60)	0.9036	0.9460	0.8612	0.0737	0.0430	0.1045
20 (µg/kg/day) + ML ($N_{\text {sub}}$ = 60)	0.9137	0.9478	0.8795	0.0748	0.0469	0.1027
40 (µg/kg/day) ($N_{\text {sub}}$ = 80)	0.9440	0.9799	0.9082	0.0531	0.0251	0.0810
80 (µg/kg/day) ($N_{\text {sub}}$ = 100)	0.9498	0.9907	0.9088	0.0424	0.0107	0.0742
Mean performance	0.9278	0.9661	0.8894	0.0610	0.0314	0.0906
Dataset 2^15^						
0.15 (µg/kg/day) ($N_{\text {sub}}$ = 50)	0.9737	0.9854	0.9619	0.0561	0.0229	0.0893
0.15 (µg/kg/day) + ML ($N_{\text {sub}}$ = 50)	0.9723	0.9843	0.9603	0.0600	0.0227	0.0975
1.5 (µg/kg/day) ($N_{\text {sub}}$ = 50)	0.9770	0.9879	0.9661	0.0458	0.0222	0.0694
1.5 (µg/kg/day) + ML ($N_{\text {sub}}$ = 50)	0.9712	0.9851	0.9573	0.0718	0.0241	0.1196
15 (µg/kg/day) ($N_{\text {sub}}$ = 50)	0.9786	0.9884	0.9687	0.0369	0.0195	0.0543
15 (µg/kg/day) + ML ($N_{\text {sub}}$ = 50)	0.9697	0.9833	0.9561	0.0718	0.0325	0.1113
150 (µg/kg/day) ($N_{\text {sub}}$ = 10)	0.9797	0.9898	0.9696	0.0153	0.0133	0.0172
150 (µg/kg/day) + ML ($N_{\text {sub}}$ = 10)	0.9682	0.9821	0.9543	0.0735	0.0219	0.1250
Mean performance	0.9738	0.9860	0.9616	0.0540	0.0224	0.0856
Dataset 3^16^						
4 N ($N_{\text {sub}}$ = 40)	0.9733	0.9845	0.9622	0.0408	0.0176	0.0639
8 N ($N_{\text {sub}}$ = 46)	0.9723	0.9840	0.9607	0.0501	0.0221	0.0781
Mean performance	0.9728	0.9842	0.9614	0.0455	0.0199	0.0710
All datasets						
Mean performance	0.9631	0.9811	0.9345	0.0548	0.0248	0.0846

Extreme cases further highlight the model’s generalization. For example, in Dataset 1^14^, the DSC for PTH 80 µg/kg/day combined with mechanical loading was 0.9498, with 0.9907 for the cortical compartment and 0.9088 for the trabecular compartment. The corresponding HD95 values were 0.0424, 0.0107, and 0.0742, respectively. This performance underscored the model’s ability to handle significant changes in bone morphology.

We presented the segmentation results for the secondary datasets [37–40] in Table 3.For example, the average DSC for Dataset 4^37^ was 0.9620, with 0.9963 for the cortical compartment and 0.9270 for the trabecular compartment. The HD95 values were 0.0342, 0.0071, and 0.0612, respectively. However, the segmentation results for human stem cell implants in young mice (8 weeks old) were lower, with an average DSC of 0.76908 and an HD95 of 0.13652. This outcome is due to the extremely young and nondense bone structure of the ovariectomized mice, which is very different from that of the mature control mice used for training.

**Table 3:** Quantitative evaluation of DBAHNet segmentation performance for the secondary datasets. The results include Dice Similarity Coefficient (DSC) and 95th percentile of Hausdorff Distance (HD95) for both cortical (C) and trabecular (T) compartments. $N_{\text {sub}}$ represents the total number of subsets, with each scan divided into 10 subsets along the z-axis.

Datasets	Avg DSC	DSC C	DSC T	Avg HD95	HD95 C	HD95 T
Dataset 4^37^ ($N_{\text {sub}}$ = 40)	0.9620	0.9963	0.9270	0.0342	0.0071	0.0612
Dataset 5^38^ ($N_{\text {sub}}$ = 26)	0.7691	0.8773	0.6609	0.1365	0.0935	0.1796
Dataset 6^39^ ($N_{\text {sub}}$ = 37)	0.9494	0.9754	0.9233	0.0155	0.0104	0.0156
Dataset 7^40^ ($N_{\text {sub}}$ = 40)	0.9110	0.9731	0.8489	0.0215	0.0154	0.0278
All datasets						
Mean performance	0.8979	0.9555	0.8395	0.0520	0.0311	0.0710

#### Qualitative evaluation

**Fig. 1:**
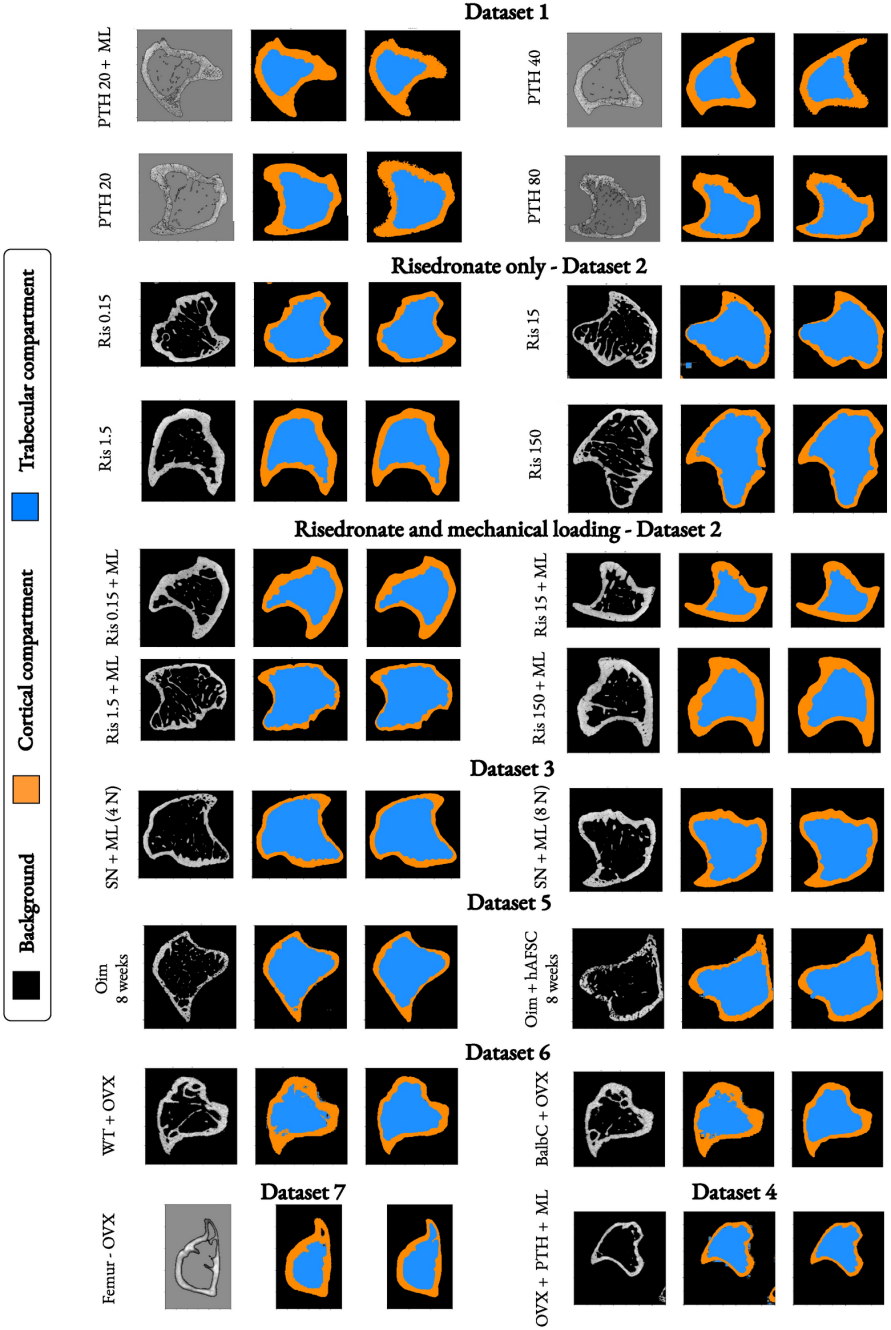
Segmentation results on the main datasets^14–16^ and secondary datasets^37–40^ under different conditions (age, resolution, medical treatment, mechanical loading, and mouse strain). The cortical compartment is shown in orange, and the trabecular compartment is shown in blue. For each sampled scan from a group in a dataset, the input processed image is shown on the left, the automatic segmentation in the middle, and the manual segmentation on the right.

We conducted a visual inspection of the entire region of interest of the bone, from the middle of the metaphysis to the proximal region of long bones near the growth plate. Figure 1 displays qualitative segmentation results for all the datasets. The visual inspection showed that the segmentation of the cortical and trabecular compartments is highly accurate, with a smooth transitional region between the two compartments. We also observed that our approach produces a much cleaner segmentation compared to semi-automatic manual segmentation, which involves interpolation and often leads to small segmentation errors in the interpolated cross-sectional slices. The segmentation images obtained under different experimental conditions demonstrated the model’s ability to delineate complex bone structures. For high doses of PTH and risedronate, visual inspection revealed precise segmentation even in regions with significant porosity. A qualitative comparison of DBAHNet with the current gold standard, the dual threshold method^12^, for four different dose groups is shown in the supplementary materials. The dual threshold method fails to properly segment the trabecular compartments in certain regions for the PTH-treated groups from Dataset^14^ (see Supplementary Fig. S1).

#### Performance across various treatments

Our model’s proficiency extended across different treatments, highlighting its adaptability. Specifically, under high PTH and risedronate treatments, the model accurately segmented bone structures, maintaining high DSC and low HD95 values. The mean DSC for Dataset 1^14^, which involves iPTH and mechanical loading, was 0.9278, with 0.9661 for the cortical compartment and 0.8894 for the trabecular compartment. For Dataset 2^15^, which involves risedronate, the mean DSC was greater at 0.9738, with 0.9860 for the cortical compartment and 0.9616 for the trabecular compartment. This difference can be attributed to the greater anabolic effect of PTH on bone, which leads to increased porosity and merging between cortical and trabecular bone, compared to the anticatabolic effect of risedronate. These differences underscored the model’s ability to generalize across different treatment regimens, handling significant morphological changes induced by these treatments. Similarly, for Dataset 3^16^, which involves mechanical loading combined with sciatic neurectomy, the mean DSC was 0.9728, with 0.9842 for the cortical compartment and 0.9614 for the trabecular compartment. These findings indicated that the model effectively handled the structural changes induced by the combined mechanical and neurosurgical interventions. For Dataset 4^37^, which involved ovariectomized mice under PTH treatment and mechanical loading, the model achieved a mean DSC of 0.9620, with 0.9963 for the cortical compartment and 0.9270 for the trabecular compartment. The HD95 values were 0.0342, 0.0071, and 0.0612, respectively. These results highlighted the model’s robustness in segmenting bone structures affected by hormonal changes and physical interventions, further demonstrating its versatility and effectiveness across various medical treatments.

#### Evaluation across different resolutions, ages, and mouse strains

The model exhibited strong performance across different resolutions, particularly excelling at the 5 µm resolution, which aligned with the training resolution of the control set. We also observed success at other unseen resolutions, such as 10.4 µm and 13.7 µm, recorded from both in-vivo and ex-vivo of different µCT scanners, further highlighting the model’s robustness. Specifically, for datasets with 10.4 µm resolution (Dataset 4^37^ and Dataset 6^39^), the average DSCs for the cortical and trabecular compartments were 0.9620 and 0.9494, respectively, and the HD95 values were 0.0342 and 0.0155, respectively, indicating good segmentation performance. The model also performed well across a range of ages, from 8 weeks to 24 weeks. Dataset 5^38^), which involves homozygous oim mice characterized by fragile and deformed bones and a very young age of 8 weeks, characterized by low bone density and active growth, achieved an average DSC of 0.7691 and an HD95 of 0.1365. Although this value was lower than that of other datasets, it still indicated good performance, particularly given the young age and genetic disorders associated with osteogenesis imperfecta. In contrast, Dataset 6^39^, involving BALB/c mice aged 24 weeks, presented an average DSC of 0.9494 and an average HD95 of 0.0155, indicating strong segmentation performance despite the age difference. Additionally, the model demonstrated strong performance across different mouse strains, showing its ability to generalize beyond the strain used for training. Dataset 6^39^, which included BALB/c mice, presented an average DSC of 0.9494 and an average HD95 of 0.0155, comparable to those of the C57BL/6J strain used in other datasets. This adaptability to different resolutions, ages, and mouse strains, including C57BL/6J, BALB/c, and homozygous oim mouse strains, suggests the model’s potential to be generalized across rodents with similar anatomical features, enhancing its utility in diverse experimental settings.

#### Adaptability to different bone types

Notably, the model, which was originally trained on control tibia scans, demonstrated the ability to generalize to a different bone type, the femur. This observation underscores the model’s comprehensive understanding of anatomical features, enabling it to adapt to the unique characteristics of new bone types. This versatility contributed to the model’s applicability in a wide range of bone segmentation tasks. Specifically, for Dataset 7^40^, which involved femur scans (OVX) at a resolution of 13.7 µm, the model achieved an average DSC of 0.9110, with 0.9731 for the cortical compartment and 0.8489 for the trabecular compartment. The corresponding HD95 values were 0.0216, 0.0154, and 0.0278, respectively. These results highlighted the model’s robust performance in segmenting femur bones, despite being trained only on tibia scans.

#### Generalization over unseen data

The model’s capacity to generalize over new, unseen data under different conditions-including variations in age, species, bone type, and experimental medical treatments-was excellent. When trained solely on control scan data, the model’s broad generalization ability emphasized its ability to function effectively in practical preclinical cases. This highlighted the power of deep learning models in providing accurate results across a spectrum of unforeseen scenarios and the model’s proficiency in training on a limited dataset, capturing both long-range dependencies and local features within the 3D volume. This comprehensive understanding allowed the model to recognize and generalize over unseen data recorded under very different experimental setups.

### Impact of postprocessing on segmentation performance

In this section, we evaluated the impact of the postprocessing module on segmentation performance. The postprocessing was applied to the high-dose drug treatment groups: 40 µg/kg/day and 80 µg/kg/day in Dataset 1^14^, as well as risedronate alone at 150 µg/kg/day and in combination with mechanical loading (ML) in Dataset 2. The results are summarized in Table 4.

**Table 4:** Quantitative evaluation of DBAHNet segmentation performance with and without post-processing across the high-dose groups in Dataset 1^14^, Dataset 3^15^ and Dataset 2^16^. The metrics include the average Dice Similarity Coefficient (DSC) and the average 95th percentile of Hausdorff Distance (HD95).

	Dataset 1^14^				Dataset 2^15^				Dataset 3^16^			
	PTH40		PTH80		Ris150		Ris150 + ML		4N		8N	
	DSC	HD95	DSC	HD95	DSC	HD95	DSC	HD95	DSC	HD95	DSC	HD95
No post-proc.	0.9440	0.0531	0.9498	0.0424	0.9797	0.0153	0.9682	0.0735	0.9733	0.0408	0.9723	0.0501
Post-proc.	**0.9489**	**0.0430**	**0.9671**	**0.0250**	**0.9821**	**0.0080**	**0.9838**	**0.0093**	**0.9791**	**0.0099**	**0.9794**	**0.0473**

Best performances are in bold.

Overall, the postprocessing step improved segmentation performance across all datasets and groups, as indicated by an increase in the DSC and a reduction in the HD95. The most significant improvement was observed in the 80 µg/kg/day PTH group, where the DSC increased from 0.9498 to 0.9671, and in the risedronate + ML group, with an increase in DSC from 0.9682 to 0.9838.

These results demonstrated the ability of postprocessing to enhance segmentation performance, particularly in groups showing the strongest anabolic response, such as those treated with PTH or subjected to mechanical loading. This highlights the potential of postprocessing to further refine the performance of deep learning models for segmenting cortical and trabecular compartments in µCT scans of mouse tibiae. The results may be further improved by fine-tuning the parameters of the postprocessing pipeline for each specific dataset or incorporating additional image processing steps tailored to the characteristics of the dataset.

### Model configuration study

In this study, we evaluated the performance of our proposed architecture by varying key parameters such as the embedding dimension $C$ and the reduction embedding vector $E$. The embedding dimension $C$ defines the size of the vector space into which the input features are mapped. Increasing $C$ generally enhances the model’s ability to capture complex patterns, but it also increases computational complexity. The reduction embedding vector $E$ specifies the downsampling ratio for the feature maps along the $x$, $y$, and $z$ dimensions in the patch embedding block. The results, summarized in Table 5, show that increasing $C$ improves the DSC, indicating better segmentation performance. Specifically, the configuration with $C = 96$ and $E = [4, 4, 2]$ achieves the highest DSC of 0.9872. However, this improvement comes with increased model complexity, as indicated by the greater number of parameters and GFLOPS. Conversely, smaller embedding dimensions reduce computational demands but also slightly decrease performance. The choice of $E$ also impacts the model’s performance and efficiency, with $E = [4, 4, 2]$ offering a good balance between accuracy and computational load. Furthermore, we demonstrated that a lighter version of the model, with reduced embedding dimensions and downsampling ratios, still achieves excellent performance. This suggested that our architecture can be adapted for environments with limited computational resources while maintaining high segmentation accuracy.

**Table 5:** Ablation study of the proposed architecture on the tibia µCT validation dataset. Abbreviations: $C$ (embedding dimension), $E$ (patch embedding downsampling Vector), GFLOPS (giga floating point operations per second).

Model configuration	Avg DSC	N Params (M)	GFLOPS
$C = 48$, $E = [4, 4, 4]$	0.9826	17	66.9
$C = 48$, $E = [4, 4, 2]$	0.9814	17.12	83.6
$C = 96$, $E = [4, 4, 4]$	0.9855	67.8	186.4
$C = 96$, $E = [4, 4, 2]$	0.9872	67.9	260

### Ablation study

We conducted an ablation study to analyze the impact of various components of our proposed architecture. An ablation study systematically removes or alters components of a model to understand each component’s contribution to the overall performance. We tested the following configurations:

1. *Configuration 1* DBAHNet without a bottleneck, which uses a standard convolution block (3D convolution, batch normalization, and GeLU activation) in both the encoder and decoder.
2. *Configuration 2* DBAHNet with the Channel-wise Attention-Based Convolution Module (CACM) in both the encoder and decoder, without a bottleneck.
3. *Configuration 3* DBAHNet with the CACM in the encoder and the Spatial-Wise Attention-Based Convolution Module (SACM) in the decoder, without a bottleneck.
4. *Configuration 4* The full DBAHNet with all the components.

Table 6 presents the results of this ablation study, highlighting the importance of each component in our architecture. The baseline configuration (Configuration 1), which uses standard convolution blocks, achieves a DSC of 0.9487. Introducing CACM to both the encoder and decoder (Configuration 2) significantly improves the DSC to 0.9846, highlighting the effectiveness of attention mechanisms in improving feature representation. Applying CACM in the encoder and SACM in the decoder (Configuration 3) results in the highest DSC of 0.9876, indicating the complementary advantages of these modules. The full DBAHNet, which includes the bottleneck, achieves a DSC of 0.9872, closely matching the performance of Configuration 3. This suggested that while the inclusion of a bottleneck does not significantly improve performance, it still contributes to feature encoding for the decoder and helps prevent overfitting.

**Table 6:** Component ablation study of the proposed architecture on the tibia µCT validation dataset. Configuration descriptions: Configuration 1—DBAHNet with no bottleneck and using standard convolution in both the encoder and decoder; Configuration 2—DBAHNet using the Channel-wise Attention-Based Convolution Module (CACM) in both the encoder and decoder, still without a bottleneck; Configuration 3—DBAHNet with the CACM in the encoder and the Spatial-Wise Attention-Based Convolution Module (SACM) in the decoder, still without a bottleneck; Configuration 4—The full DBAHNet with all the components. Abbreviations: GFLOPS (Giga Floating Point Operations Per Second).

Model configuration	Avg DSC	N Params (M)	GFLOPS
Configuration 1	0.9487	58.3	325.6
Configuration 2	0.9846	77.9	258.8
Configuration 3	**0.9876**	62.2	258.8
Configuration 4 (Full DBAHNet)	0.9872	67.9	260

Best performance is in bold.

## Discussion

The results from our study demonstrated the significant ability of DBAHNet to segment cortical and trabecular bone compartments from high-resolution µCT scans of the mouse tibia. By employing our large diverse dataset summarized in Table 7, we ensured a robust evaluation across a multitude of conditions, including variations in resolution, age, strain, drug treatments, surgical procedures, and mechanical loading. This diversity provided a comprehensive landscape for assessing the generalizability of our model.

A key strength of our study is the model’s ability to generalize effectively, even though it was trained exclusively on a limited set of control scans. This underscored the potential of deep learning models to be trained on restricted datasets while maintaining high performance across diverse and unseen scenarios. DBAHNet achieved excellent segmentation accuracy not only on the control dataset but also across various challenging conditions where bone morphology was significantly altered due to drug treatments, surgical procedures, or mechanical loading. Moreover, our automated approach outperformed the manual and semiautomatic algorithms used to label the ground truth, which involve manual segmentation of sampled slices followed by interpolation. The semiautomatic method often results in noisy interpolated slices, as segmentation quality decreases with fewer manually segmented slices. In contrast, our model performs segmentation of the basis of the characteristic features of the two types of bone compartments, resulting in more accurate, faster, and smoother segmentation.

Importantly, the computational complexity of DBAHNet requires considerable computational resources for training. This highlights the need for adequate hardware to fully leverage the model’s potential. Additionally, the use of high-resolution 3D µCT scans necessitates significant data storage and processing power, potentially posing challenges for large-scale studies. These issues can be mitigated by employing a lighter version of the DBAHNet model, which we have previously demonstrated to be functional, reducing the input cropping size, or decreasing the complexity of the architecture by reducing the sequence length of the transformer, as employed in P2T^42^.

Notably, the model’s performance on Dataset 5^38^ was relatively lower than that of the other datasets, primarily because of the undeveloped young mice (8 weeks of age), which are models of the Oim mouse strain, exhibit skeletal deformities, fractures, and cortical thinning. This dataset exhibited an average DSC of 0.7691, highlighting the limitation of training a deep learning model on a limited set of control scans and expecting it to generalize effectively across extreme scenarios. This underperformance underscores the necessity of training on a larger and more varied dataset to capture the full spectrum of bone morphology variations. While the model’s performance is robust, it is still subject to the quality and diversity of the training data. Scenarios with extreme deviations from the control dataset, such as very young or old mice or highly specialized medical treatments not included in the training set, may yield less accurate results. Expanding the training dataset to include a wider variety of conditions could enhance the model’s robustness and generalizability.

Future improvements will focus on retraining the model on a more comprehensive and varied dataset that includes a broad spectrum of conditions. We are currently collecting a comprehensive dataset and preparing manual labeling for the final preparation of our last model. This model, trained on a large comprehensive dataset, will be integrated into our automated pipeline for µCT mouse tibia assessment. This approach aims to develop a truly robust and accurate model capable of segmenting the cortical and trabecular compartments of µCT scans of the mouse tibia under any preclinical experimental setup. Additionally, by integrating our deep learning model into our global segmentation pipeline, we can refine the segmentation results and address the limitations posed by the variability of diverse preclinical animal experiments. The global segmentation pipeline ensures a general improvement in the segmentation results of our deep learning model, and prepares the cortical and trabecular compartments for the subsequent steps of morphological and statistical analysis.

In summary, our study validated the effectiveness of DBAHNet in achieving high segmentation performance across diverse scenarios. Despite these challenges, our work highlighted the exciting potential of DBAHNet and the global pipeline to transform bone segmentation tasks. By expanding and diversifying the training dataset in future work, we anticipate creating an even more robust and generalizable model. This will advance our understanding of bone structure and significantly improve segmentation accuracy in preclinical and experimental settings, highlighting the large impact of deep learning in high-resolution biomedical imaging.

This segmentation module is part of a larger project aimed at developing an automated end-to-end pipeline for analyzing the microarchitecture of the mouse tibia using high-resolution µCT scans in a preclinical setting. The goal of this pipeline is to provide fast, accurate, and fully automated analysis of the effects of various experimental conditions-such as drug treatments, surgical interventions, mechanical loading, and aging-on bone remodeling and dynamics. This will help accelerate research in biomechanics and improve our understanding of bone remodeling and diseases like osteoporosis.

## Methods

### Datasets

**Fig. 2:**
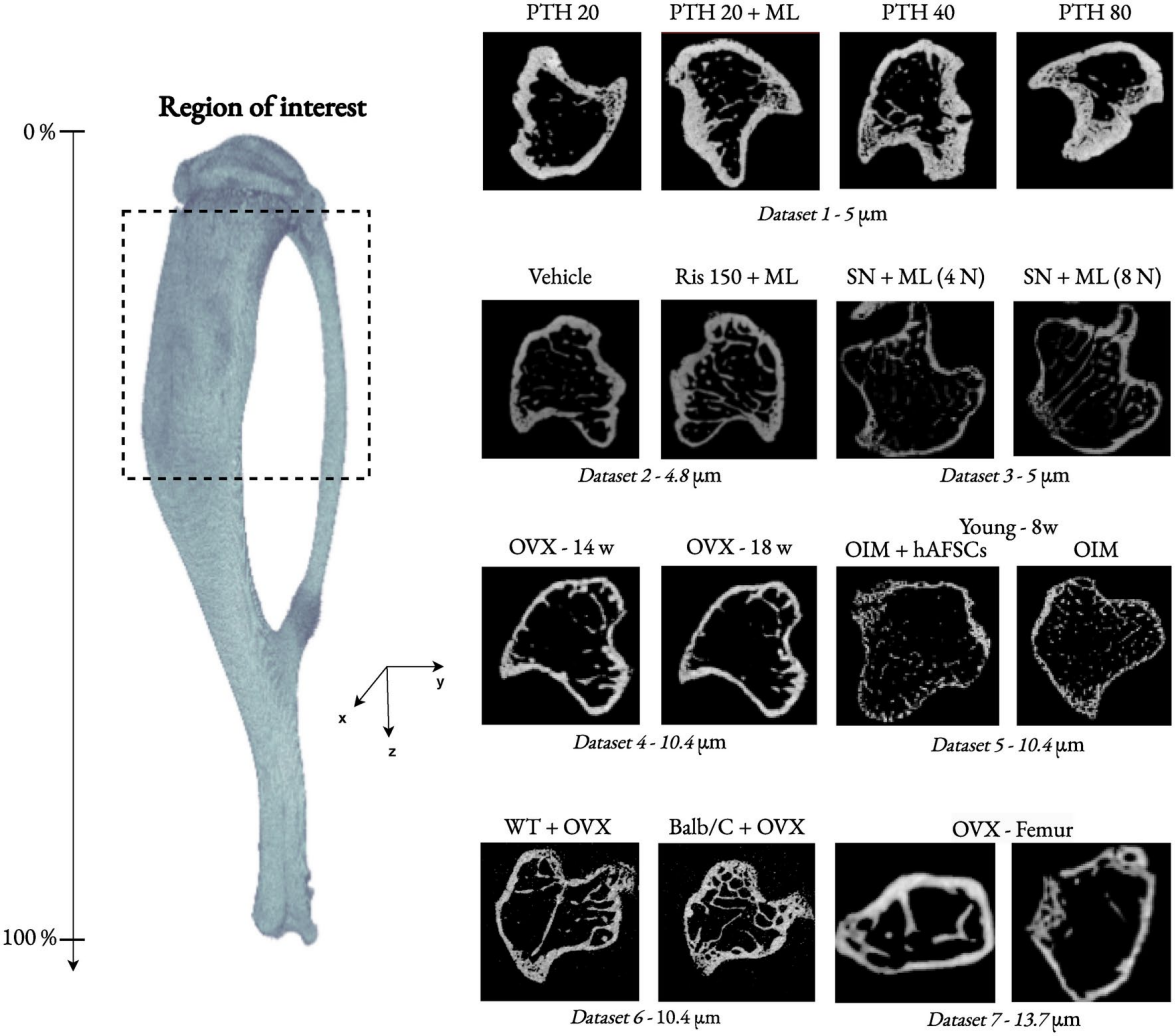
Large, diversified collection of high-resolution µCT scans at the proximal region of interest of the mouse tibia, covering multiple experimental setups^14–16,37–40^. These setups include different treatments and conditions, such as parathyroid hormone (PTH), risedronate (Ris), sciatic neurectomy (SN), ovariectomy (OVX), human amniotic fluid stem cells (hAFSC), mechanical loading (ML), age (8 to 24 weeks), mouse strain (C57BL/6, BALB/c, and homozygous oim), and scanning resolution (4.8 µm to 13.7 µm).

We evaluated the effectiveness of our deep learning segmentation architecture, DBAHNet, across various experimental studies (see Fig. 2). Our extensive dataset contains a total of 163 tibia scans derived from seven experimental studies^14–16,37–40^. These scans exhibit varied bone morphology due to differences in scanning resolutions, mouse strains, ages, drug treatments, surgical procedures, and mechanical loading. The dataset includes four mouse strains: C57BL/6, BALB/c, C57BL/6JOlaHsd, and homozygous oim, focusing on young and mature animals ranging from 8 to 24 weeks of age. These animals received a variety of treatments, including ovariectomy (OVX), human amniotic fluid stem cells (hAFSC), sciatic neurectomy (SN), risedronate (Ris), and parathyroid hormone (PTH) treatments at different doses. Additionally, some studies have applied mechanical loading (ML) to investigate the individual and combined effects of these treatments on bone structure. The mouse tibiae were imaged via µCT at different resolutions ranging from 4.8 µm to 13.7 µm. This high resolution enabled a detailed assessment of trabecular and cortical bone structures. The dataset covered various responses to drug interventions, with mechanical loading experiments designed to mimic physiological stress and explore bone adaptation responses.

We manually segmented the scans following standard guidelines^10^ by sampling sectional 2D slices with a fixed step tailored to the specific bone region. This involved manual segmentation of both the cortical and trabecular compartments at these specific cross-sectional slices. We subsequently employed the Interpolation Biomedisa platform^11^ for semiautomated segmentation, which uses weighted random walks for interpolation, and considers both the presegmented slices and the entire original volumetric image data. We performed postprocessing on the interpolated 3D labels to smooth and remove outliers, followed by visual inspection to validate the final ground truth labels.

The datasets used in this study are described as follows:

- *Control dataset*^14–16^ This dataset is based on three separate studies and includes tibiae from C57BL/6 virgin female mice aged 19–22 weeks. It comprises 74 control tibiae that were not subjected to any treatments in the referenced preclinical experiments. High-resolution µCT scans were performed via SkyScan 1172 (SkyScan, Kontich, Belgium), with resolutions ranging from 4.8 µm to 5 µm (ex vivo).
- *Dataset 1*^14^ This study investigated the impact of the bone anabolic drug intermittent PTH on bone adaptation in virgin female C57BL/6 mice. The treatment doses used were 20, 40, and 80 µg/kg/day, both alone and in combination with ML. The dataset includes images of four groups: PTH 20 (N=6), PTH 20 + ML (N=6), PTH 40 (N=8), and PTH 80 (N=10), all aged 19 weeks. Images were captured at a resolution of 5 µm (ex vivo). Both mechanical loading and PTH treatment have anabolic effects on bone, promoting bone formation and increasing bone mass. Their combined effects result in more pronounced anabolic responses, further complicating segmentation due to increased bone remodeling and porosity, especially near the growth plate.
- *Dataset 2*^15^ This study examined the effects of the anticatabolic drug risedronate on bone adaptation in virgin female C57BL/6 mice. The dataset includes three risedronate dose groups (0.15, 1.5, and 15 µg/kg/day) with and without mechanical loading, each with $N=5$ samples, and a risedronate 150 µg/kg/day group with one loaded and one nonloaded sample (N = 1), all aged 19 weeks. Images were captured at a resolution of 4.8 µm (ex vivo). This segmentation is challenging because of the effects of the anticatabolic risedronate and the anabolic effect of ML. Compared with the control, risedronate reduces bone resorption, resulting in greater trabecular bone volume and trabecular number, whereas ML increases trabecular and cortical.
- *Dataset 3*^16^ This study assessed the impact of mechanical loading on bone adaptation in C57BL/6 mice subjected to right sciatic neurectomy to minimize natural loading in their right tibiae. The dataset includes images of two groups, 4 N (N=5) and 8 N (N=5), aged 20 weeks. Images were captured at a resolution of 5 µm (ex vivo). The segmentation challenges arise from localized bone loss due to neurectomy and the subsequent anabolic bone changes induced by mechanical loading.
- *Dataset 4*^37^ This study provides high-resolution in vivo µCT images of tibiae from female C57BL/6 mice subjected to OVX, which mimics postmenopausal osteoporosis characterized by increased bone remodeling, followed by combined PTH (100 µg/kg/day) and ML interventions. The dataset includes wild-type (WT) female C57BL/6 OVX (N=4) mice recorded at weeks 14, 18, 20, and 22. Images were captured at a resolution of 10.4 µm (in vivo). OVX increases porosity and bone remodeling, presenting significant segmentation challenges. The combination of PTH and ML further complicates segmentation due to their anabolic effects, enhancing bone formation and altering bone architecture. Additionally, the changes in the resolution and age of the mice compared with those in the control dataset complicate the generalization of segmentation techniques.
- *Dataset 5*^38^ This study conducted high-resolution µCT analysis of bone microarchitecture in 8-week-old homozygous oim mice treated with human amniotic fluid stem cells (hAFSC). The dataset includes images of the Oim (N=3) and Oim + hAFSC (N=3) groups. Images were captured at a resolution of 5 µm (ex vivo). Osteogenesis imperfecta (OI) is characterized by severe characteristics, such as reduced size, skeletal fragility, frequent fractures, and abnormal bone microarchitecture, in OIM mice. Treatment with hAFSC improved bone strength, quality, and remodeling. The young age of the mice, combined with their deformed shape due to the nature of the mouse strain (homozygous oim) and hAFSC treatment effects, presents significant segmentation challenges. Their bones are not fully mature and are less dense, complicating the generalization of segmentation techniques from the control dataset of untreated mature bones.
- *Dataset 6*^39^ This study explored the impact of ovariectomy on bone structure and density in female C57BL/6 and BALB/c mice by comparing the WT and OVX groups. The dataset includes four groups: C57BL/6 WT (N=1), C57BL/6 OVX (N=1), BALB/c WT (N=1), and BALB/c OVX (N=1). Images were captured at a resolution of 10.4 µm (in vivo) at the age of 24 weeks. The differences in the structure of the strains (C57BL/6 and BALB/c), OVX bone loss effects, and lower resolution make it difficult to generalize segmentation techniques from the control dataset.
- *Dataset 7*^40^ This study focused on a murine model of osteoporosis in C57BL/6JOlaHsd OVX female mice. The dataset includes images of femurs from C57BL/6JOlaHsd female mice (N = 4) that underwent OVX at the age of 14 weeks. Images were captured at a resolution of 13.7 µm (ex vivo) at the age of 17 weeks. Compared with tibiae, the combination of femur bones, which have different structural characteristics, a much lower resolution of 13.7 $\mu$m, and OVX-induced bone loss presents substantial segmentation challenges.

This diverse dataset encompasses a wide range of conditions, including different ages, strains, resolutions, drug treatments, surgical procedures, and mechanical loading, providing a rich resource for a robust validation of our deep learning model. A summary of the datasets used in this study is presented in Table 7. The main datasets^14–16^ are our own extensive collections, which contain very high-resolution µCT scans at 5 µm. The secondary datasets^37–40^ consist of publicly available samples collected to test the segmentation under new unseen experimental conditions.

The datasets used in this study were obtained from independent experiments conducted by their respective institutions. For Dataset 1^14^ and Dataset 2^15^, all procedures complied with the UK Animals (Scientific Procedures) Act 1986, with ethical approval from the ethics committee of The Royal Veterinary College (London, UK). Dataset 3^16^ was similarly approved by the ethics committee of the University of Bristol (Bristol, UK). Dataset 4^37^ followed the ARRIVE guidelines and was approved by the local Research Ethics Committee of the University of Sheffield (Sheffield, UK). Dataset 5^38^ was conducted under UK Home Office project licence PPL 70/6857, and Dataset 6^39^ under project licence PPL 40/3499, both overseen by the University of Sheffield. Finally, Dataset 7^40^ received approval from the Local Ethical Committee for Animal Research of the University of the Basque Country (UPV/EHU, ref M20/2019/176), adhering to European Directive 2010/63/EU and Spanish Law RD 53/2013. All original studies ensured compliance with relevant ethical guidelines, and our use of these datasets strictly followed their established approvals.

**Table 7:** Summary of the datasets used in this study. The control dataset is listed first (Underline), followed by the main datasets (Bold) and secondary datasets (Italic). Abbreviations: parathyroid hormone (PTH), mechanical loading (ML), risedronate (Ris), sciatic neurectomy (SN), ovariectomy (OVX), human amniotic fluid stem cells (hAFSC), wild type (WT). $N$ represents the number of µCT mouse tibia scans.

Dataset	Treatment	N	Resolution (µm)	Age (weeks)	Mouse strain
Control Dataset^14–16^	Control (None)	74	4.8–5 (ex vivo)	19–22	C57BL/6
**Dataset 1**^14^	**PTH, ML**	**30**	**5 (ex vivo)**	**19**	**C57BL/6**
**Dataset 2**^15^	**Risedronate, ML**	**31**	**4.8 (ex vivo)**	**19**	**C57BL/6**
**Dataset 3**^16^	**SN, ML**	**10**	**5 (ex vivo)**	**20**	**C57BL/6**
*Dataset 4*^37^	*OVX, PTH, ML*	*4*	*10.4 (in vivo)*	*14–22*	*C57BL/6*
*Dataset 5*^38^	*hAFSC*	*6*	*5 (ex vivo)*	*8*	*homozygous oim*
*Dataset 6*^39^	*OVX*	*4*	*10.4 (in vivo)*	*24*	*C57BL/6, BALB/c*
*Dataset 7*^40^	*OVX (femur)*	*4*	*13.7 (ex vivo)*	*17*	*C57BL/6JOlaHsd*
Total		**163**			

The region of interest in the mouse tibia used in this research was cropped from the metaphysis, starting just below the growth plate (approximately 6–8% of the bone length from the proximal region, where trabecular bone is highly present and active) and extending to approximately 60–65% of the bone length. Additionally, the control scans were obtained from a slightly deeper region within the metaphysis, where trabecular bone is not excessively present in the medullary area. The reason for choosing this deeper region is to detect any potential trabecularization of the cortical bone or growth of the trabecular bone, which can occur under certain conditions such as with drug treatments or aging.

### General segmentation pipeline

**Fig. 3:**
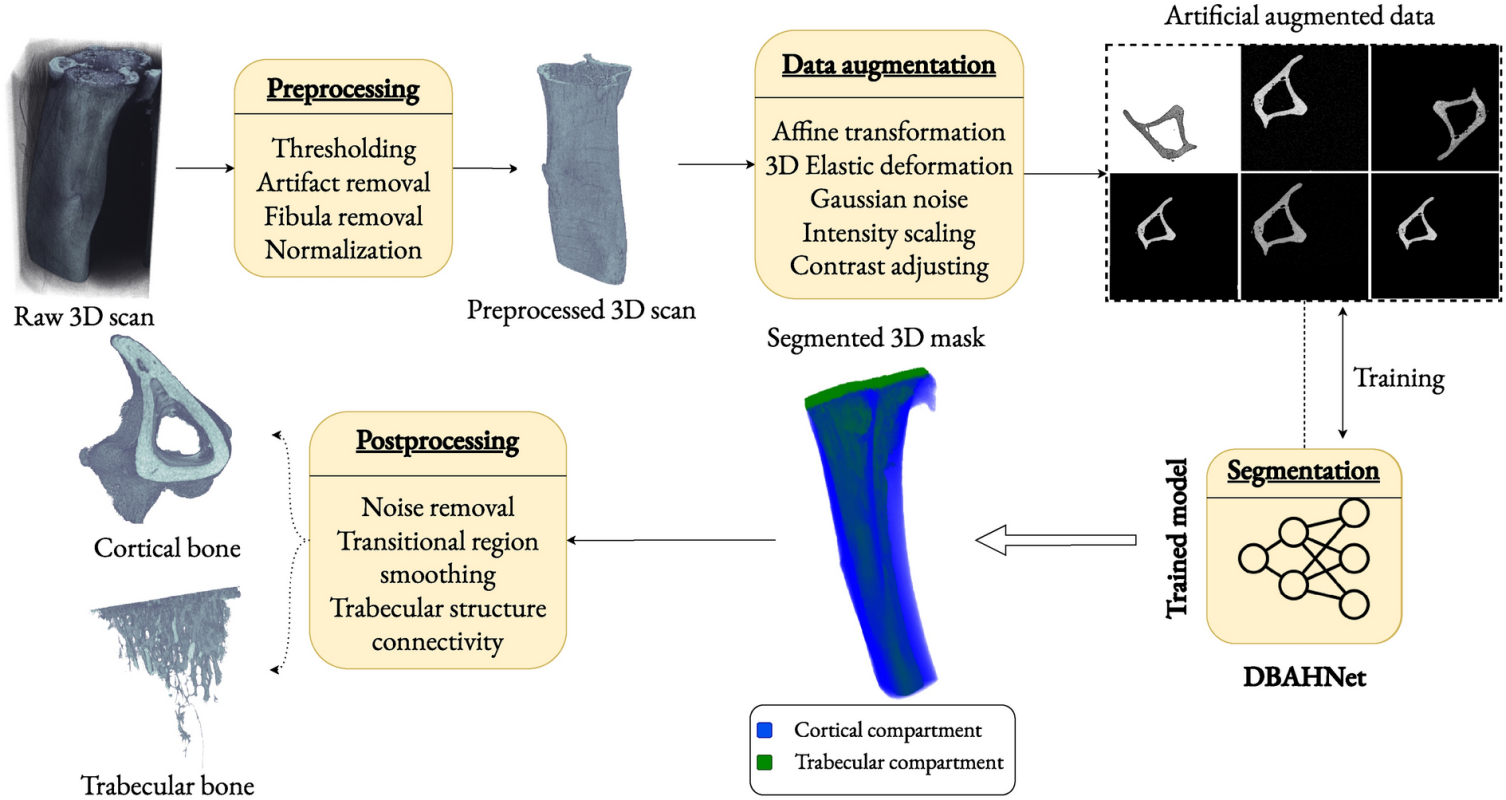
The global pipeline for robust 3D high-resolution µCT mouse tibia segmentation. The pipeline includes preprocessing, data augmentation, segmentation via DBAHNet, and final postprocessing.

This section describes our automated, robust, deep learning-based pipeline developed for 3D high-resolution µCT segmentation, that specifically targets the cortical and trabecular compartments of the mouse tibia, as illustrated in Fig. 3. The general segmentation pipeline begins with preprocessing the raw 3D µCT scans via image processing techniques to isolate the mouse tibia. These preprocessed scans serve as the input for training the deep learning model. During training, data augmentation automatically expands the dataset, creating variations that improve model accuracy. The model is trained iteratively on the training set, with continuous monitoring of the validation mean Dice score and loss until convergence is achieved. After training, the model produces segmentation masks for both the cortical and the trabecular compartments. A postprocessing step further refines the segmentation to enhance the extraction of the cortical and trabecular bone. The detailed steps of the pipeline are outlined below.

#### Preprocessing

We subjected the raw 3D µCT scans to a series of preprocessing steps to prepare the data for segmentation. Thresholding: We applied the Otsu thresholding algorithm^43^ to automatically separate the bone from the background, which includes experimental materials such as the sample holder and the resin. To ensure the retention of actual bone voxels, particularly for the trabecular bone, a threshold margin of $M = 5$ was subtracted from the threshold obtained by the algorithm to maintain connectivity. Artifact removal: We eliminated any remaining noise by retaining the largest connected component, which represents the bone. These two preprocessing steps are crucial, as they not only clean the bone from the experimental background, allowing the model to focus on segmenting the cortical and trabecular compartments, but also significantly reduce the size of the µCT scans. Working with 3D µCT scans at very high resolution requires careful consideration of efficiency, as training complex deep learning architectures becomes computationally demanding with larger input data. Performing these steps substantially reduces the size of the input images. For instance, a raw scan of the full mouse tibia recorded at 5µm from Dataset 1^14^ is approximately 2.4 GB. After background removal and autocropping, the file size is reduced to approximately 150 MB (both sizes are reported for the compressed Nifti format). Fibula removal: We removed the second-largest component, representing the fibula, at each cross-sectional slice along the $z$-axis. Normalization: We normalized the voxel values via z-score normalization, transforming the image intensities so that the resulting distribution has a mean of zero and a standard deviation of one. The z-score normalization is defined as $Z = \frac{X - \mu }{\sigma }$, where $Z$ is the normalized intensity value, $X$ is the original intensity value, $\mu$ is the mean intensity value of the image, and $\sigma$ is the standard deviation of the intensity values of the image.

#### Data augmentation

To enhance the model’s generalization ability, we employed various data augmentation techniques applied to the original 3D scans during each batch generation throughout the training. Random affine transformations: We applied rotations and scaling to simulate changes in the orientation and scale of the bone relative to the scanner. The rotation range is $[0, \pi ]$ along the $z$-axis, and the scaling factor range is $s \in [0.85, 1.25]$. 3D Elastic deformations: We introduced nonlinear distortions to mimic natural bone variability via the following formula: $x' = x + \alpha \cdot {\mathcal {G}}(\sigma )$, where ${\mathcal {G}}(\sigma )$ is a random Gaussian displacement field with a standard deviation $\sigma \in [9, 13]$ and magnitude $\alpha \in [0, 900]$. Random Gaussian Noise: We added random Gaussian noise to simulate varying scanner qualities. The noise addition is given by $x' = x + {\mathcal {N}}(0, \sigma ^2)$, where ${\mathcal {N}}(0, \sigma ^2)$ is Gaussian noise with zero mean and variance $\sigma ^2 = 0.1$. Random intensity scaling: We scaled the intensity of the images to account for differences in imaging conditions. The intensity scaling is given by $x' = x \cdot (1 + f)$, where the scaling factor $f$ ranges from $-0.1$ to $0.1$. Random contrast adjusting: We adjusted the contrast of the images to account for differences in imaging conditions. The contrast adjustment is expressed as $x' = x^{\gamma }$ with $\gamma \in [0.5, 4.5]$. These transformations ensure the robustness and accuracy of the deep learning model by providing diverse and realistic variations in the training data. This approach generates new, artificially augmented data during training, where data augmentation is applied live to each batch with a small probability ($p = 0.1$), simulating scans under different experimental setups for the training of our deep learning model.

#### Segmentation

In this study, we employed a novel deep neural network architecture, DBAHNet (dual branch attention-based hybrid network), which was previously validated by comparing its performance with popular state-of-the-art architectures on the control dataset^36^. DBAHNet is specifically designed for high-resolution 3D µCT bone image segmentation, and focuses on the cortical and trabecular compartments. This architecture advances deep learning approaches by integrating both transformers and convolutional neural networks to effectively capture local features and long-range dependencies. The hybrid design of DBAHNet leverages the ability of convolutional layers for local feature analysis and the attention mechanism of transformers. In this work, we apply DBAHNet within a comprehensive pipeline to evaluate its robustness across various conditions and datasets, demonstrating its utility beyond the initial conference presentation. The complete architecture of DBAHNet is detailed in the subsequent sections.

#### Postprocessing

The final phase involved applying postprocessing techniques to increase the quality of the segmentation masks and mitigate the inherent imperfections in the segmentation process: Noise removal: We removed any segmentation noise and outliers by retaining the largest connected component. Transitional region smoothing: We used morphological opening filters to remove small openings at the endosteum surface of the cortical bone and assign them to the trabecular bone. The morphological opening filter is defined as: $\text {Opening}(A, B) = (A \ominus B) \oplus B$, where $A$ is the set of foreground voxels in the binary image, $B$ is the structuring element (a sphere with radius $K_o$), $\ominus$ denotes the erosion filter, which removes pixels from the boundaries of objects, eliminating small openings at the endosteum surface, and $\oplus$ denotes the dilation filter, which adds pixels to the boundaries of objects, restoring the original size of the cortical surface while maintaining a smooth transition to the trabecular bone. The kernel value $K_o$ is set to 3. Trabecular structure connectivity: We ensured the connectivity of the trabeculae for accurate morphometry in subsequent steps. For this, we perform Connected Component Analysis by identifying and labeling all connected components in the binary mask of the trabecular bone and merging components that are close to each other. Merging is performed via a morphological closing filter with a kernel radius $R_c = 1$, corresponding to the minimum distance required to merge disconnected trabeculae. The morphological closing filter can be defined as follows: $\text {Closing}(A, B) = (A \oplus B) \ominus B$, where $A$ is the set of foreground voxels in the binary image, $B$ is the structuring element (a sphere with radius $R_c$), $\oplus$ denotes the dilation filter, which adds pixels to the boundaries of objects, potentially bridging small gaps caused by segmentation errors, and $\ominus$ denotes the erosion filter, which removes pixels from the boundaries of objects, and restores the original object size while maintaining new connections. The different modules of the general segmentation pipeline facilitated the extraction and subsequent morphological analysis of both cortical and trabecular bone from three-dimensional µCT scans, enabling their visualization and assessment of their respective morphological parameters for preclinical skeletal studies.

### Architecture of DBAHNet

**Fig. 4:**
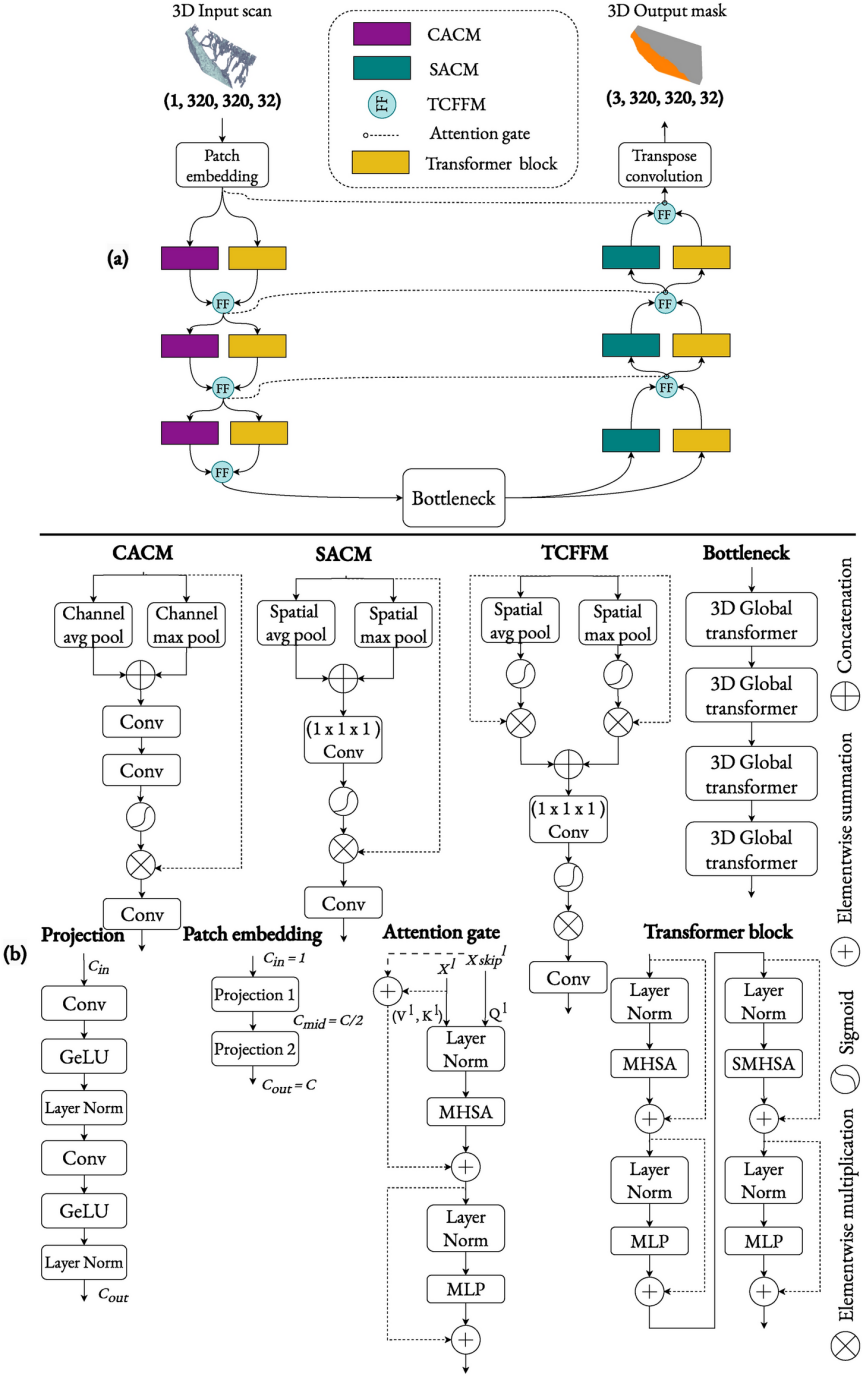
(**a**) Global architecture of the dual-branch attention-based hybrid network (DBAHNet) for 3D µCT mouse bone tibia imaging segmentation. (**b**) Diagram of all internal modules of DBAHNet, including the channel-wise attention-based convolution module (CACM), the spatial-wise attention-based convolution module (SACM), the transformer-convolution feature fusion module (TCFFM), the bottleneck, the patch embedding block, the attention gate, and the transformer block.

The proposed architecture, the Dual-Branch Attention-based Hybrid Network (DBAHNet), features a dual-branch hybrid design that incorporates both convolutional neural networks (CNNs) and transformers in the encoder and decoder pathways (see Fig. 4A). The patch embedding block projects the 3D scan into an embedding space with $C = 96$ channels via successive convolutions. This process results in a reduced-dimensionality space, defined by the reduction embedding vector $E = [4, 4, 4]$, creating a patch embedding of size $(C, \frac{H}{4}, \frac{W}{4}, \frac{D}{4})$, where $H$, $W$, and $D$ represent the height, width, and depth of the input 3D scan, respectively. This embedding serves as the input to both the transformer and convolutional branches, each consisting of three hierarchical levels.

In the encoder pathway, each level comprises two sequential Swin transformers blocks in the transformer branch and a Channel Attention-Based Convolution Module (CACM) in the convolution branch. The transformer branch uses 3D-adapted Swin transformers to process feature maps at multiple scales, capturing global long-range dependencies within the volume. Each transformer block consists of two layers; the first employs regular volume partitioning, whereas the second uses shifted partitioning to increase the connectivity between layers. In the convolution branch, the CACM enhances cross-channel interaction by concatenating the outputs of global average pooling and maximum pooling, followed by two GeLU-activated 3D convolutions to create an attention map. This map modulates the initial feature map through elementwise multiplication, and a final 3D convolution further encodes the output for subsequent layers.

The outputs from the transformer and convolution branches at each level are fused via the Transformer-Convolution Feature Fusion Module (TCFFM). The TCFFM performs downsampling in the encoder by applying channelwise average pooling to $x_{\text {Tr}}$ and $x_{\text {C}}$ (the feature maps from the transformer and convolution branches), followed by a sigmoid function to generate an attention mask that filters the channels. The results are then concatenated and encoded through a 3D convolution layer. After encoding, the resulting feature maps are downscaled to $(8C, \frac{H}{32}, \frac{W}{32}, \frac{D}{32})$ and passed to the bottleneck. The bottleneck consists of four global 3D transformer blocks that perform global attention over all the downsampled feature maps, aggregating information to provide a comprehensive representation for the decoder.

The decoder mirrors the encoder symmetrically. It uses the spatial attention-based convolution module (SACM) instead of the CACM to enhance relevant spatial features for focused reconstruction of the segmentation mask. The SACM applies max-pooling and average-pooling, concatenates the results, and uses a $1 \times 1 \times 1$ convolution to create an attention map. This attention map modulates the input feature map, which is further processed by a final 3D convolution. The TCFFM module in the decoder performs upsampling, restoring the original volume size. Throughout the decoder, feature maps from all layers are filtered via attention gates and residual skip connections from the encoder. Finally, a transpose convolution reconstructs the segmentation masks. All internal components of DBAHNet are illustrated in Fig. 4B.

#### Transformer block

We leveraged a 3D adaptation of Swin transformers^32^, which perform self-attention within a local volume of feature maps at each hierarchical level to capture enriched contextual representations of the data. Each transformer unit consists of two consecutive transformers. The first transformer employs regular volume partitioning, whereas the second transformer introduces shifted local volume partitioning to ensure connectivity with the preceding layer’s local volumes. For a given layer $l$, the input ${\textbf{x}}^{l-1}$ first undergoes layer normalization (LN) and is then processed by a multihead self-attention (MHSA) mechanism. The output of the MHSA is added to the original input via a residual connection, resulting in the intermediate output $\hat{{\textbf{x}}}^l$. Next, $\hat{{\textbf{x}}}^l$ is normalized again and passed through a multilayer perceptron (MLP), with another residual connection to produce the output ${\textbf{x}}^l$. The second transformer, which uses shifted partitioning, applies a shifted multihead self-attention (SMHSA) mechanism. This shifted transformer increases the connectivity between layers. The normalized output ${\textbf{x}}^l$ from the previous step is processed by the SMHSA with a residual connection, resulting in the intermediate output $\hat{{\textbf{x}}}^{l+1}$. Finally, $\hat{{\textbf{x}}}^{l+1}$ undergoes another normalization and passes through an MLP, with a residual connection to yield the output ${\textbf{x}}^{l+1}$. The Swin transformer block is expressed by the system of equations in Eq. (4).

4 $$\begin{aligned} \begin{aligned} \hat{{\textbf{x}}}^l&= \text {MHSA}\left( \text {LN}\left( {\textbf{x}}^{l-1}\right) \right) + {\textbf{x}}^{l-1}, \\ {\textbf{x}}^l&= \text {MLP}\left( \text {LN}\left( \hat{{\textbf{x}}}^l\right) \right) + \hat{{\textbf{x}}}^l, \\ \hat{{\textbf{x}}}^{l+1}&= \text {SMHSA}\left( \text {LN}\left( {\textbf{x}}^l\right) \right) + {\textbf{x}}^l, \\ {\textbf{x}}^{l+1}&= \text {MLP}\left( \text {LN}\left( \hat{{\textbf{x}}}^{l+1}\right) \right) + \hat{{\textbf{x}}}^{l+1} \end{aligned} \end{aligned}$$

The self-attention mechanism is computed using Eq. (5).

5 $$\begin{aligned} \text {Attention}(Q,K,V) = \text {Softmax}\left( \frac{QK^T}{\sqrt{d_k}}\right) V \end{aligned}$$

Here, $Q$, $K$, and $V$ represent queries, keys, and values, respectively, and $d_k$ is the dimension of the key and query.

#### Channel-wise attention-based convolution module (CACM)

In the encoder, we utilized a convolution unit based on channelwise attention, assigning distinct levels of importance to different channels, thereby enhancing feature representation. Let $x \in {\mathbb {R}}^{C \times H \times W \times D}$ be the input feature map. We first apply both global average pooling and maximum pooling channelwise, yielding a $\left( C, 1, 1, 1\right)$ vector, which is then concatenated. This concatenated vector undergoes a 3D convolution to an intermediate dimension, resulting in a $\left( \frac{C}{2}, 1, 1, 1\right)$ size, followed by a GeLU activation function. This output is further processed through a second 3D convolution to restore the original channel dimension. An attention map is subsequently generated via a sigmoid activation function, which is then elementwise multiplied with the initial feature map, modulating it on the basis of channelwise attention. Finally, a third convolution is applied, downsampling the dimensions to $\left( 2C, \frac{H}{2}, \frac{W}{2}, \frac{D}{2}\right)$, to be used in subsequent layers.

#### Spatial-wise attention-based convolution module (SACM)

In the decoder, we employed a convolution module that ensures spatial attention; this module focuses selectively on the salient features and regions during the reconstruction of the segmentation mask, aiding in the preservation of detailed structures and enhancing accuracy. Let $x$ be the input feature map such that $x \in {\mathbb {R}}^{C \times H \times W \times D}$. Initially, we apply both max-pooling and average-pooling to extract two robust feature descriptors. These descriptors are concatenated along the channel axis before undergoing a $1 \times 1 \times 1$ convolution to yield a feature map of dimensions $(1, H, W, D)$. Next, a sigmoid activation function derives the attention map, which is then elementwise multiplied with the original input to obtain a feature map of dimensions $(C, H, W, D)$. Considering the necessity of upsampling the feature maps during the decoding phase, a transpose 3D convolution operation with a stride of 2 is utilized to upsample the features, resulting in the final feature maps of dimensions $\left( \frac{C}{2}, 2H, 2W, 2D\right)$.

#### Transformer-convolution feature fusion module (TCFFM)

In the TCFFM block, the feature maps obtained from both the transformer and convolution pathways, denoted as $x_{\text {Tr}}$ and $x_{\text {C}}$, each belonging to the space ${\mathbb {R}}^{C \times H \times W \times D}$, are fused at each hierarchical level. Here, $H$, $W$, and $D$ represent the dimensions of the feature maps, and $C$ is the number of channels. Initially, channel-wise average pooling is applied to $x_{\text {Tr}}$ and $x_{\text {C}}$ to extract a representative value for each channel of the feature maps. These values are transformed into weights using a sigmoid function, generating an attention mask that enhances significant channels and suppresses less relevant channels. The results are subsequently concatenated and passed through a downsampling convolution layer, followed by a local-volume transformer block, to perform the fusion and leverage the combined strengths of both pathways in the subsequent layers.

#### Bottleneck

In the bottleneck, we reduced the dimensionality of the resulting feature maps from the encoder and employ a series of four global 3D transformer blocks, similar to those used in the Vision Transformer (ViT)^31^. These blocks perform global attention over all the downsampled feature maps. They excel at aggregating information from the entire feature map, enabling an understanding of the global context and providing a comprehensive representation to the decoder.

#### Attention gate

Instead of using regular concatenation in the skip connections such as those in U-Net^17^, we employed attention gates (AGs)^30^ to enhance the model’s ability to focus on target structures of varying shapes and sizes. Attention gates automatically learn to suppress irrelevant regions in an input image while highlighting salient features relevant to a specific task.

Specifically, the output of the $l^e$-th TCFFM of the encoder, $X_{l}^e$, is transformed via a linear projection into a key matrix $K_l^e$ and a value matrix $V_l^e$. This transformation encodes the spatial and contextual information necessary for the attention mechanism. The output feature maps after the $l^d$-th upsampling layer of the TCFFM in the decoder, denoted $X_{l}^d$, serve as the query $Q_l^d$. We apply one layer of the transformer block to $Q_l^d$, $K_l^e$, and $V_l^e$ in the decoder, computing self-attention as previously described for the transformer block.

## Supplementary Information

Supplementary Information.

## Data Availability

The main µCT datasets used in this study are not publicly available. The secondary µCT datasets were collected from publicly accessible sources. The model and code developed for this study are publicly available at https://github.com/bigfahma/DBAHNetGitHub. The trained weights of DBAHNet are available upon reasonable request from the corresponding authors.
