# Supplementary material for: A robust deep learning approach for segmenting cortical and trabecular bone from 3D high resolution µCT scans of mouse bone
Source: Sci Rep. 2025 Mar 13;15:8656. doi: 10.1038/s41598-025-92954-1 (PMC11906900; doi:10.1038/s41598-025-92954-1)
Supplement: Supplementary file 1 — Supplementary Information. [file 41598_2025_92954_MOESM1_ESM.pdf]

Robust and generalized deep learning-based approach for mouse bone segmentation from 3D high-resolution CT imaging data

Amine Lagzouli[1,2,*], Peter Pivonka[1], David M.L. Cooper[3], Vittorio Sansalone[2], and Alice Othmani[4,**]

[1]School of Mechanical, Medical, and Process Engineering, Queensland University of Technology, Brisbane, Australia
[2]Univ Paris Est Créteil, Univ Gustave Eiffel, CNRS, UMR 8208, MSME, F-94010 Créteil, France
[3]Department of Anatomy, Physiology, and Pharmacology, University of Saskatchewan, Saskatoon, Saskatchewan, Canada
[4]Université Paris-Est Creteil (UPEC), LISSI, Vitry sur Seine 94400, France
[*]aminelagzouli02@gmail.com
[**]alice.othmani@u-pec.fr

Supplementary Information

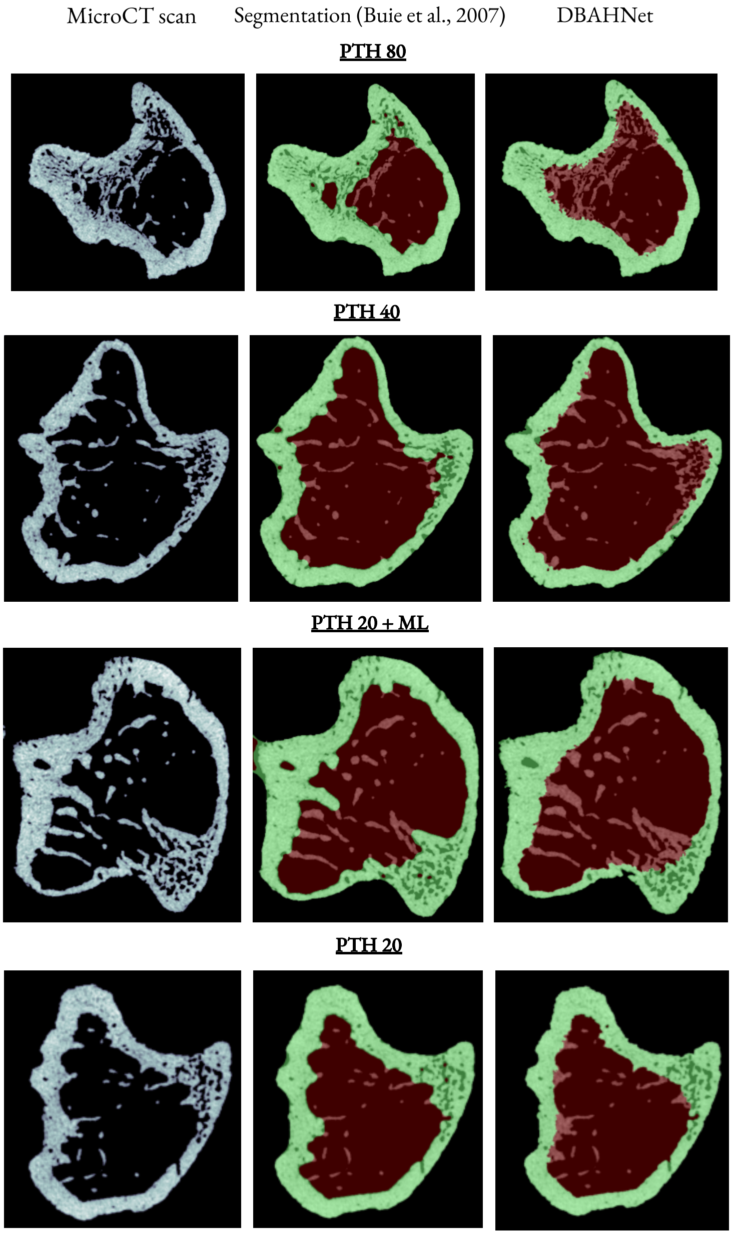

Supplementary Figure S1: Segmentation results of the cortical and trabecular compartments in the mouse tibia with our proposed approach (DBAHNet) and the dual threshold method [12] on Dataset 1 [14]. The cortical compartment is shown in green, and the trabecular compartment is shown in red. For the displayed scans, the input image is on the left, the dual threshold segmentation is in the middle, and DBAHNet segmentation is on the right.
